# Integration of omics: more than the sum of its parts

**DOI:** 10.1186/s40170-016-0143-y

**Published:** 2016-02-19

**Authors:** Joerg Martin Buescher, Edward M Driggers

**Affiliations:** Vesalius Research Center, VIB, Leuven, Belgium; Department of Oncology, KU Leuven, Leuven, Belgium; General Metabolics, 30 Arlington Street, Winchester, MA 01890 USA

**Keywords:** Metabolomics, Proteomics, Transcriptomics, Genomics, Data integration, Metabolic network, Cancer, Cellular physiology, Algorithm

## Abstract

Genome scale data on biological systems has increasingly become available by sequencing of DNA and RNA, and by mass spectrometric quantification of proteins and metabolites. The cellular components from which these -omics regimes are derived act as one integrated system in vivo; thus, there is a natural instinct to integrate -omics data types. Statistical analyses, the use of previous knowledge in the form of networks, and the use of time-resolved measurements are three key design elements for life scientists to consider in planning integrated -omics studies. These design elements are reviewed in the context of multiple recent systems biology studies that leverage data from different types of -omics analyses. While most of these studies rely on well-established model organisms, the concepts for integrating -omics data that were developed in these studies can help to enable systems research in the field of cancer biology.

## Background

Our ability to acquire highly multivariate datasets (“omics” datasets) on biological systems has increased tremendously in recent years. Advances in high-throughput DNA sequencing, RNASeq methods, and various advances in mass spectrometry, all coordinated with computational and algorithmic advances, have progressively lowered the barrier to accessing -omics data. This situation is a tremendous advance and often leads directly to a challenging question: how can I integrate these -omics to construct a systems-level understanding?

The instinct to integrate -omics datasets with each other is natural for systems biologists: various “-omics” regimes of course derive from an integrated whole in whatever biological system they originate. Modern molecular research has revealed that important insight can be found not only within those regimes (at the genomic, epigenomic, transcriptional, protein, post-translational modification, and metabolic levels) but also through understanding interactions between these regimes [1]. In this short review, we first summarize a number of recent multi-omics studies in cancer and then proceed to focus on a set of representative integrated -omics case studies outside of cancer biology. These non-cancer studies provide a general framework from which to consider the design of integrated -omics studies, without the constraint of viewing the problem as a cancer-specific challenge. We feel this approach will lead to better designs in cancer-specific studies and is justified by the longer history of integrated studies in areas outside of cancer research. We attempt to focus on reported studies that employ accessible algorithms and to provide a general approach to integrated -omics studies that will benefit both biologists and informaticians. Where possible, we categorize studies as either network creation studies seeking to inform a new network (often between -omics levels), or network function studies, seeking to understand existing networks more fully and accurately (often within -omics levels). The paragraphs that follow organize around three fundamental design challenges for biologists who run -omics integration experiments: first, the need to understand the statistical behavior of each -omics independently prior to integration; second, the need to account for relationships between the specific layers of biology that are being compared in the integrated study; and finally, the explicit challenge to be aware of timing differences within and between the -omics domains.

## Multi-omics studies in cancer

Multiple cancer-related studies have been published in recent years that use more than one type of -omics data set, including metabolomics data. Multi-omics cancer data sets are frequently “co-analyzed”—each -omics analyzed independently using statistics and then compared graphically or qualitatively—rather than “integrated,” by which we mean to indicate that the multiple -omics have been merged into a single mathematical, statistical representation of biological behavior; both approaches have strengths and limitations. For example, one popular approach to co-analyzing -omics datasets is enrichment analysis [2–6]. This technique evaluates the statistical overrepresentation of a priori-assigned gene ontology (GO) terms among a group of metabolites, genes, or enzymes that are found to be different between conditions or between cancer tissue and neighboring healthy tissue. Because the same GO terms have been used for metabolites, genes, and proteins, they can be used to establish links across -omics data sets. However, the power of this approach is limited by the uncertainty and incompleteness of the a priori GO term association, which varies between -omics types. Because GO terms are built on existing network information, studies whose primary analyses are through GO list membership comparisons are generally network function studies that enrich our knowledge of existing networks more fully.

A second frequently used approach to co-analyzing multi-omics data has been to plot the independently generated results onto a known (metabolic) network [3–5, 7, 8], which is also a network function study approach. While these visualizations can be very helpful to develop and communicate ideas, they are typically drawn manually and are inherently prone to interpretation, as any (network-based) visualization is a trade-off between clarity, completeness, and the use of established patterns (for example, the convention of drawing TCA cycle below glycolysis). There is no one-size-fits-all solution to this challenge, and the optimum of this trade-off is influenced by the author’s interpretation of the data. Consequently, visualization cannot replace unbiased, statistical data analyses but should rather be seen as a useful way for communicating ideas.

A range of software tools are available for the network-based visualization of -omics data, many of which can also handle multiple types of -omics data. Commercial products include Ingenuity Pathway Analysis [9], which can link customer data to build-in canonical pathways, and Omix [10], which is particularly strong for metabolic networks and comes with some modeling capabilities. Powerful open-source solutions include Vanted [11], which can very nicely display multiple datasets simultaneous on the same map, and MetScape [12], which builds onto the popular Cytoscape software suite. Inherently platform independent are online tools like Prometra [13] and Paintomics [14].

An example where the systematic and unbiased statistical integration of -omics data sets has been achieved by the correlation of metabolites and genes across a set of biopsy samples from pancreatic tumors and neighboring healthy tissue, reported by Zhang et al. [6]. This study utilized a weighted co-expression network analysis routine to identify clusters of metabolites that responded similarly across the patient cohort. Working from these lists of coordinately produced metabolites, the authors utilized a pathway enrichment analysis (using IPA software) to assign the metabolites to known GO terms, enabling direct comparison with transcripts sharing GO membership. The authors identified eight metabolites and four enzymes from lipid metabolism as potential therapeutic targets. While these were not the only hits from the statistical data analysis, the known involvement in the same part of metabolism presumably led the authors to focus on these targets. Note that the initial use of WGCNA, followed only afterward by GO mappings, makes this a network creation study, which has the potential to discover new pathway relationships.

In a separate study also using metabolomics and transcriptomics from patient samples, data were first analyzed separately and later liked through shared annotations [15], however, here with the goal of identifying predictive biomarkers for breast cancer. As the same pathways were independently identified in the respective -omics, the combination of both analyses mutually supported each other.

In some contexts, the known network relationships within one biological regime, or even between regimes, can help with phenotype classification. For example, an improved classification of breast cancer tissue was achieved by combining transcriptomics and metabolomics data [16]. The authors used the Spearman correlation between transcripts and each of eight metabolite fold-changes in 34 breast tumors to identify functionally related entities. Thus, the combined dataset is not only larger than either single -omics data set, enabling a more finely grained resolution for classification, but it inherently contains independently collected information connecting the two levels, revealing information beyond that suggested purely by a comparably larger single -omics dataset.

The following studies differ from those mentioned above in focusing on biological areas outside of cancer, and for that reason, we have organized them according to broad experimental design challenges, rather than according to biological findings at the systems level. The biological findings are mentioned mostly as examples for what kind of insight can be obtained by various approaches to -omics data integration. Nevertheless, the design challenges highlighted here apply equally to all systems-level multi-omics studies.

## Design challenge 1: understand the statistical behavior of the readouts from each -omics regime independently and in detail

**Fig. 1:**
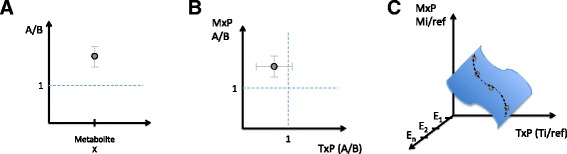
The number of perturbations and the number of -omics levels determine the possible read-outs. The number of biological states, or perturbations, that are compared in a multi-omics study define the type of information that can be learned. **a** Graphical depiction of the relative read-out of a single value from an individual -omics study (here, a metabolite) in biological states A and B. **b** Relative read-out from an integrated -omics study comparing two biological states A and B (using metabolomics (*MxP*) and transcription (*TxP*) in this graphic), where each axis is the relative signal of treatment vs. control for each separate -omics regime. These data can be used to improve understanding of existing networks for the system in these two states; however, it cannot provide information about the general relationships between the molecules measured in the two -omics readouts. **c** Relative integrated read-out from a multi-state experiment, where *E*_*1*_-*E*_*i*_ represent the different states (e.g., perturbations or time points). These integrated data can potentially reveal a new network capturing the relationships between the metabolites and transcripts of the integrated -omics readouts

Many -omics studies begin with the aim of comparing two biological states: treated vs. untreated, transformed vs. untransformed, fed vs. starved, etc. A strictly two-state comparison, where one state serves as the baseline control for measurements in the other state, provides a terrific basis for network function studies that bring greater insight and confidence to existing networks but is highly limited in guiding the creation of previously unknown networks. Counterintuitively, collecting -omics data from multiple -omics types within a biological system does not guarantee that it will be possible to learn about the relationship between those -omics types; the fundamental reason for this limitation is explained in Fig. 1a–c. In contrast, when multiple perturbations are applied, creating multiple biological states for -omics comparison (one such example is time-course information; see discussion below), the resulting integrated data can be used in a network creation study to inform the creation of new networks that capture relationships between -omics regimes within the system, as depicted in Fig. 1c. However, for both categories—two-state and multi-state—a key design challenge is to understand the specific dynamic range, signal-to-noise, confidence intervals, and associated *p* values within the specific individual -omics datasets that comprise the study. Values that are sometimes imported wholesale from the heuristics of the field at large (e.g., “take signals that are >2-fold up or down”) risk seriously compromising integrated -omics research, as these statistics will guide the choice of integration method and the approach to interpretation of the findings.

Attempting to understand the simultaneous transcriptional and metabolic factors that participate in macrophage polarization, Jha et al. [17] compared the simultaneous transcriptional and metabolomics profiles in each of two different activated forms of mouse macrophage, M1 and M2, to the same unactivated M0 baseline control—in each case, a strict two-state comparison. A number of transcriptional profiling studies had been reported for this experimental system previously, providing a set of expected responses within the transcriptomics; additionally, accepted metabolite markers of polarization such as itaconic acid had been reported, along with the known networks of mammalian metabolism, and so, -omics integration was employed to provide added confidence and insight into differences in the metabo-transcriptional network between the two different states (i.e., a network function study). In order to enable the integration, using a variation of the BioNet algorithm on a global murine cellular reaction network, multiple hypothesis-corrected *p* values for each metabolite and transcript were utilized as inputs for the method (rather than bulk cut-off values). Among the novel findings of this integrated study was a metabolic break-point in the TCA-cycle of M1 macrophages at the isocitrate dehydrogenase (IDH) step, which was subsequently validated using ^13^C tracer studies. However, note that the researchers did not report any previously undiscovered network connections within or between transcription and metabolism, due to the inherent limitation captured in Fig. 1b.

A different approach was used in Askenazi et al. [18] where the specific purpose was to gain an understanding of previously undetermined network relationships connecting gene expression and secondary metabolism in the filamentous fungi *Aspergillus terreus*. In this network creation study, the researchers needed to compare the transcriptional and metabolomics read-outs among many different states in order to empirically build up the relationships that comprise the novel network (akin to Fig. 1c), where in this case the multiple perturbed states were created by transformation of the fungi using a library of approximately 400 transcription factors and so-called global regulator genes. The first step in integration was once again to acquire a clear picture of the variation, signal-to-noise, and patterning captured in each -omics regime individually (see Fig. 2 in [18]). Note that in this case, the specific data of the study was used to boot-strap statistical distributions and confidence intervals in the transcript profiling data. To integrate the two read-outs, in the absence of comparable scales for the magnitude of change in each -omics, a categorical statistical relationship, Goodman and Kruskal’s gamma [19], was chosen as the measure of association between transcription and metabolism. Applying these categorical statistics, they were able to discover the biosynthetic cluster responsible for production of the polyketide Geodin, a significant secondary metabolite in cultures of of *A. terreus*.

Note that in each case, regardless of the aim to reveal previously unknown network relationships between the different -omics vs. the aim of bolstering insight within a pre-existing network, these studies required incorporation of the statistical distributions within each -omics domain individually before the integrated conclusions could be drawn.

## Design challenge 2: non-obvious relationships exist between -omics regimes within their original biological context

**Fig. 2:**
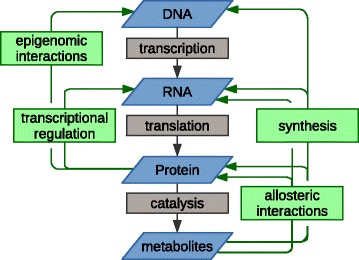
The relationship among the -omics levels beyond the central dogma of molecular biology. The relationship among the -omics (*blue*) is multifold. Enzyme catalysis and then central dogma of molecular biology are the most prominent relationships (*grey*). Additional relationships (*green*) can become important for the integration of -omics data depending on the experimental setup and time-scales analyzed

In the integration of different -omics data types, it is also extremely helpful to take into account the inherent relationship between the biological regimes represented by the respective -omics readouts. The most familiar of such relationship is the central dogma of molecular biology: genes to transcripts to protein. However, metabolomics is a functional readout that is subsequent to the central dogma framework, and metabolites can loop back into it at multiple, less familiar points (Fig. 2). For example, they serve as both substrates and products in enzymatic reactions, they are the monomers from which protein and RNA are synthesized, they can allosterically regulate the activity of enzymes or the folding or RNA molecules, and they can allosterically regulate the activity of transcription factors and thus indirectly the expression of multiple genes. Thus, the process of integrating -omics is an effort to disentangle multiple cycles of functional relationships, rather than an effort to reveal linear connections. Described below are just a few cases where less familiar relationships between levels have been revealed through integrated -omics studies or else used to enhance the strength of conclusions from integrated studies.

The relationship between individual enzymes and their substrates and products can be described by the well-established equations for enzyme kinetics. Linking these equations for the enzymes and metabolites of one pathway into one unified set of differential equations has been achieved [20, 21]. However, the strongly non-linear behavior of these equations, particularly when connected into pathway-level systems, and the challenge of handling these by numerical computations have greatly limited the extension of kinetic models to the genome scale. Despite this limitation, understanding general features of the relationship between enzymes and metabolites at the genomic level is highly desirable when considering the integration of proteomic and metabolomic datasets.

This relationship between metabolite and enzyme levels has been studied experimentally in *Saccharomyces cerevisiae* by combing standardized enzyme kinetics and statistical analysis with multi-omics integration [22]. In this network function study, hypotheses were validated against a comprehensive collection of transcriptional, proteomic, and metabolomic datasets on *S. cerevisiae* in which levels of enzymes were experimentally perturbed. The resulting data validated previous observations that metabolite concentrations are on the order of the k_M_ values [23] and further provided observation of an inverse correlation of metabolite and enzyme fold changes upon alteration in enzyme abundance. Moreover, it was shown that perturbations to single enzymes trigger only local alteration in metabolite abundance. The practical conclusion is that metabolomics data can be used as a read out for enzyme activity that is indirect yet covers multiple enzymes simultaneously; the fundamental biological conclusion is that metabolic networks realize a trade-off between minimizing the abundance of enzymes and of metabolite that enables maintenance of flux homeostasis despite perturbations, without the need for transcriptional regulation. The correlation of transcripts (as surrogates for enzyme activity) and metabolites was subsequently generalized and termed Concentration Change Coupling Analysis in an independent report from Zelezniak et al. [24].

The flow of small molecule metabolites into the assembly of macro-molecules such as proteins and poly-nucleic acids represents a trans-omics relationship that may become a factor in some experimental designs as well. The impact of the obvious fact that RNA is synthesized from metabolites was demonstrated for the response of *S. cerevisiae* to a sudden relief of glucose limitation [25]. The authors observed a disproportionally fast degradation of transcripts from genes that were downregulated in response to relief of glucose limitation. These were demonstrated to result from mRNA degradation as a nucleotide salvage pathway that served to stabilize intracellular nucleotide concentrations in the face of the increased nucleotide triphosphate demand for glucose catabolism in the overall regulatory response.

## Challenge 3: capitalize on time resolution in -omics data

**Fig. 3:**
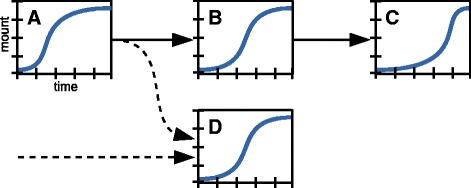
Time-resolved data informs pathway directionality. A schematic model for the interpretation of time course data. The observed time courses of the levels of *A*, *B*, and *C*, which are known to belong to the same linear pathway reveal the order of the three components in the pathway and the direction of the flux through the pathway. *A*, *B*, and *C* can be metabolites in a metabolic pathway, phosphorylated proteins in a kinase cascade, or members of any other matter- or signal-transducing pathway. The component *D*, for which the network connection to the *ABC* pathway is unknown, cannot be upstream of *A*, and it cannot be downstream of *C*. This information can be valuable in validating hypotheses on the network connection of *D*

The sequence of time-dependent responses at different -omics levels can serve as an extra dimension of data that can be considered in designing a biological experiment for integrated -omics. In general, external perturbations trigger a sequence of events that contain information about the connectivity and the directionality of underlying biological networks (Fig. 3). This additional layer of information is based on the simple logic that a cause must precede its effect. Time-resolved (dynamic) -omics data sets can therefore serve as a basis to construct new biological networks and to unravel which network connections are determined for a given systems response (note the consistency with the need for multi-state comparisons, as described above). Ideally, samples for time-resolved studies can be collected at intervals that match the dynamics of the molecular system. Assessing the metabolomics response to metabolic and post-translational perturbations typically requires sampling on a seconds time scale [26, 27]; the inherent dynamics of the transcriptional regulation network requires sampling over the course of several minutes to hours. A baseline observation can be obtained simply by initiating the sequence of sampling in the unperturbed state.

The response to a physiological perturbation such as the addition of drugs or nutrients to a cell culture triggers an interconnected metabolic and regulatory response. Multiple dynamic -omics data sets are required to capture the full extent of the cellular response. In addition, their integration allows the study of the crosstalk between the -omics—both of which were achieved in a comprehensive study of integrated cellular response by Buescher and colleagues [28]. The authors observed the dynamic response to the addition of nutrients by metabolomics, transcriptomics, and proteomics in *Bacillus subtilis*. Samples for different -omics analyses were taken at intervals ranging 20 s to 150 min (two doubling times for *B. subtilis*) following nutrient perturbations, starting from the same homogeneous culture. Parallel experiments with over 200 strains expressing GFP reporters for promoter activity complement the data set. Post-transcriptional regulation was detected by a dynamic model correlating the time courses of promoter activity with transcript abundance, and transcripts with protein levels, respectively, where the simple analysis relies on whether the transcript abundance of a gene matches the activity of its promoter or the abundance of its protein in time. The observation of a matching time course for related elements across -omics levels is the most frequent situation. However, the authors detected 110 cases of statistically significant mis-matches in time across the datasets, which indicate that additional regulation events occurred during or after transcription of the respective genes. In each case, additional experimentation was required to elucidate the specific regulatory mechanism underlying a specific mismatch.

For example, a surprisingly slow response to the availability of glucose was observed: the maximum glucose uptake rate was realized after about one doubling time, and metabolite time profiles hinted towards a bottleneck in glucose utilization upstream of phosphoenolpyruvate. Next, intra- and extra-cellular metabolomics time courses were integrated into the known metabolic network to estimate time courses of the net reaction rates of all reactions in the model. The time courses of these rates were then correlated with the time courses of the abundances of the proteins that catalyze these reactions. A strong positive correlation was observed for the enzymes of two operons, which are responsible for glucose uptake and lower glycolysis, respectively; in addition, the authors demonstrated that continuous induction of either operon allows for instantaneous glucose utilization immediately after addition. Thus, despite more than half of all *B. subtilis* genes showing a significant change in expression in this experiment (when considering the endpoint alone), including consideration of time-courses in the integrated -omics study revealed two specific regulatory events that govern the cellular adaptation at the physiological level.

In network creation studies, time resolved -omics data can greatly assist in the creation of the network connecting the measured entities. A classic example of metabolite profiling, though clearly not a modern “omics” study, provides an excellent tutorial: the pioneering work accomplished by Melvin Calvin and coworkers leading to the discovery of the CO_2_ fixation pathway, now known as Calvin-cycle [29]. Starting in the mid 1940s, they subjected cultures of the unicellular green algea *Chlorella* to pulses of ^14^C labeled CO_2_ and obtained samples at up to 5-s intervals. By 2D thin layer chromatography, they then resolved the order in which the label arrives in the intermediates of the downstream metabolic pathway; and thus, the pathways of CO_2_ flow into the central carbon metabolite pools.

Modern studies frequently start with a list of candidate networks to identify the one that best fits the measured time course data. To identify the allosteric regulation that governs the glycolytic-to-gluconeogenic switch in *Escherichia coli*, Link et al. compiled 126 putative allosteric interactions between metabolites and enzymes and subjected cultures to 30-s pulses of ^13^C labeled sugars [27]. Within this short time window, protein abundances can safely be assumed to be constant, and in this context, time-resolved metabolite labeling was sufficient to identify eight enzymes that are allosterically activated or inhibited by up to four (from a total of five) different metabolites to enable the reversal of flux through one of the major carbon utilization pathways; one newly discovered allosteric interaction in this work was the activation of FBP-ase by pyruvate.

One recent study, aimed toward identification of a new network, tracked time courses for abundance of metabolites and transcripts following three different perturbations of nitrogen metabolism [30]. The authors identified metabolites lying upstream (“influencing”) or downstream (“influenced by”) of the transcription factor TORC1 in *S. cerevisiae*. This was enabled by systematic integration of time courses of metabolites and transcripts in a dynamic model and included prior knowledge of the targets of the TORC1. This work independently validated previous hypotheses on TORC1 regulation and additionally highlighted AICAR as a key regulator of amino acid and nucleotide metabolism.

In case the different sets of -omics data are available on the same time scale (or can be interpolated to a common time scale), co-clustering by K-means can identify related biological entities [31]. This was applied in a study that subjected cultures of *E. coli* to five different stress conditions and observed the cellular response by quantifying transcripts and metabolites multiple time points before and after the onset of the stress condition. With this unsupervised clustering approach, the authors could find the association of metabolites and transcript fold changes in amino acid synthesis pathways. In addition, they used canonical-correlation analysis to identify condition-dependent association between metabolites and enzymes in central carbon metabolism, and could thereby find metabolic pathways that are of particular importance in the response to stress.

## Conclusions

The integration of -omics data can yield more than the sum of the individual -omics experiments and potentially provides access to the interactions that can occur among all classes of molecules in a cell, determining cellular physiology and behavior. Many of the studies gain insight using two or more -omics data sets captured during the same biological process. This integration of multiple -omics, often captured in parallel during the same biological process, required the authors to tailor novel solutions for practical challenges in integrating -omics data for their particular research questions. For example, both Zhang et al. [6] and Fendt et al. [22] correlate metabolomics with expression data (reviewed above). On the one hand, Zhang et al. do not employ the known metabolic network as an a priori input but rather use correlation analysis to identify genes that are linked to lipid metabolism. On the other hand, Fendt et al. correlate expression values of enzymes only with the metabolites that are known to participate in the respective reactions and thus observe how flux homeostasis is preserved upon perturbations in enzyme activity.

Both the practical solutions and these fundamental biological relationships developed using model organisms can also be applied to clinically relevant fields such as cancer biology. We expect that future studies employing integrative methods will have groundbreaking impact on our understanding of cancer on a cellular and on a systemic level because they bridge the gap between genome-level data sets and the molecular mechanisms that govern cellular physiology. Consequently, molecular interactions that are based on known molecular mechanism, and not only on association with the same GO term, can then also be studied on a global scale. Time-resolved data sets are particularly suited for this approach.

The systematic, statistical analysis of data generated for the same model system is greatly facilitated by the development of shared experimental material such as the NCI60 panel of cancer cell lines [32] or the human tumor panel TCPA [33]. The widespread access to these biological resources removes the need to generate all the required -omics data within a single study. Instead, -omics data sets from the same panel can be re-analyzed in various combinations and from various perspectives and can build up on each other going forward. Historically, the field of cancer research has been particularly effective in assembling these types of open resources for the community, and we anticipate that as integrated -omics studies become more frequent in cancer research, many novel pathways and targets for therapy will be revealed.
